# Preventive Antepartum Care

**DOI:** 10.1155/S106474499400044X

**Published:** 1994

**Authors:** William J. Ledger

**Affiliations:** Department of Obstetrics and Gynecology New York Hospital/Cornell Medical Center 525 East 68th Street New York NY 10021 USA

## Abstract

As the role of the obstetrician-gynecologist evolves to include primary care, the obstetrician must assume greater responsibility for providing prenatal preventive care, particularly regarding the STORCH^5^ pathogens.


Infectious Diseases in Obstetrics and Gynecology 2:83–90 (1994)
© 1994 Wiley-Liss, Inc.

### Preventive Antepartum Care

**William J. Ledger**
*Department of Obstetrics and Gynecology, New York Hospital/Cornell Medical Center, New York, NY*

ABSTRACT

**As the role of the obstetrician-gynecologist evolves to include primary care, the obstetrician must assume greater responsibility for providing prenatal preventive care, particularly regarding the STORCH⁵ pathogens.** © 1994 Wiley-Liss, Inc.

KEY WORDS
Pregnancy, fetus, STORCH⁵ pathogens, screening

The role of the obstetrician in providing preventive care has been emphasized by the recent rediscovery by leaders of our specialty that the obstetrician-gynecologist provides primary care. This new awareness, stimulated by the Clinton health plan's emphasis on primary care, is further fueled by the realization that funding for surgical subspecialty residency slots will be markedly reduced when any portion of this plan is implemented by Congress into law. A practical result is that, if we declare ourselves to be generalists, we must take the responsibility of teaching preventive primary care. This pre-disease focus for the obstetrician in caring for the pregnant woman is emphasized in this article because the first patient encounter with the obstetrician-gynecologist often occurs when the patient is already pregnant.

The framework for this discussion of preventive care in pregnancy is based upon an expansion of the mnemonic TORCH. Originally, these 5 letters stood for the first letter of a variety of infectious agents that cause little or minimal symptomatology in the pregnant female host, but could have a serious impact upon the fetus and the newborn. The original mnemonic is translated as follows:

T *T*oxoplasmosis
O *O*ther
R *R*ubella
C *C*ytomegalovirus
H *H*erpes

This mnemonic was expanded by Gilles Monif more than a decade ago to STORCH, the German word for "stork," with the S for *S*yphilis. In the early 1990s, Maurice Druzin suggested a new mnemonic, STORCH⁵ (Table 1). This scheme added 4 new agents with an H in the name title: *H*epatitis B, *H*uman immunodeficiency virus (HIV), *H*uman papillomavirus (HPV), and *H*uman parvovirus. Human parvovirus is an especially fitting choice for the fifth H pathogen for it causes fifth disease in children, which should make it easy to remember for the practitioners reading this article.

My views presented here may, at times, represent a deviation from the stands of the American College of Obstetricians and Gynecologists (ACOG) as noted in a recent technical bulletin,[1] but they do represent what I believe is appropriate preventive care free of either economic or political considerations.

### SYPHILIS

Antepartum testing for syphilis is almost universally practiced by American obstetricians. I support this strategy despite the incongruities in the Amer-

**Address correspondence/reprint requests to Dr. William J. Ledger, Department of Obstetrics and Gynecology, New York Hospital/Cornell Medical Center, 525 East 68th Street, New York, NY 10021.**

Received March 28, 1994

Accepted July 7, 1994

**TABLE 1. Mnemonic STORCH[5]**

| | Agents |
|---|---|
| S | Syphilis |
| T | Toxoplasmosis |
| O | Other: |
| | Measles |
| | Varicella |
| | Group B streptococcus |
| | Gonorrhea |
| | Chlamydia |
| | Tuberculosis |
| R | Rubella |
| C | Cytomegalovirus |
| H[5] | Herpes |
| | Hepatitis B |
| | HIV |
| | HPV |
| | Human parvovirus |

ican philosophy of prevention of this organism compared with other known pathogens for the fetus. For example, syphilis, like toxoplasmosis, is very uncommon. Until the recent upsurge in congenital syphilis in urban centers, it was estimated in the United States to be a less frequent cause of infection than toxoplasmosis. This low frequency is not a uniform phenomenon across the country. A remarkable increase of new cases of congenital syphilis has been noted among the urban poor population of New York, Florida, Texas, and California.[2] The reappearance of a historical pathogen is part of a legacy of the trickle-down economics of the Reagan and Bush years of the 1980s and early 1990s in which there was an increase in the number of urban poor, an increase in the homeless population, and a decrease in federal funding for social services for these impoverished groups. As a result, there has been a rise in the incidence of congenital syphilis, an epidemic spawned, in large part, by the use of prostitution among urban poor women to support their cocaine habits.[3] This problem is so frequent in some urban centers that negative serum samples for serologic testing for syphilis are diluted to check for false negatives caused by a prozone phenomenon due to the high-antigen load.[4] For the average practitioner dealing with middle-class patients, syphilis remains an uncommon event and the evaluation of a patient with a positive-reagin test, which is nonspecific, is just as complicated, vague, and disconcerting as is the antibody testing for women who are antibody positive for toxoplasmosis. Specific treponemal antibody testing must be done when the reagin test is positive to confirm the diagnosis of syphilis, which is an indication for treatment. I personally believe that the difference between the almost universal testing for syphilis among American obstetricians and the rare testing for toxoplasmosis is related to medical-school and residency training, not to practice realities. American physicians understand the intricacies of syphilis testing; in contrast, they have not been trained in the serologic diagnosis of toxoplasmosis. Rather than maintain this unsatisfactory status quo, American physicians should upgrade their knowledge about toxoplasmosis testing.

There have been treatment changes for the pregnant woman suspected of having syphilis. For the patient with a history of penicillin allergy, penicillin skin testing should be carried out and she should be given penicillin in increasing doses to reach the therapeutic levels needed for cure. The older option of using erythromycin does not effectively treat the newborn.[5] Concomitant testing for HIV should be offered, for HIV-positive women need higher doses of conventional penicillin, such as procaine penicillin, 1,200,000 units daily for 10 days, to avoid the subsequent development of central nervous system (CNS) syphilis.[6] In addition, all pregnant women treated for syphilis should be closely observed at the initiation of treatment for a Jarisch-Herxheimer reaction, which, if untreated, could result in preterm labor, preterm delivery, or fetal death.[7]

### TOXOPLASMOSIS

The lack of antepartum testing for this pathogen remains to me one of the great mysteries of the 20th century. The reasons American physicians give are straightforwardly espoused without hesitation. Toxoplasmosis is a rare newborn infection, and testing for it would not be cost effective. This attitude persists despite the fact that the lowest estimate of this infection in the newborn is 1.1/1,000 births.[8] The American obstetrician's lack of awareness of the dangers of toxoplasmosis could be related to the fact that most infections are not apparent at birth but discovered later by the pediatrician as the child fails to develop properly. Another reason given for not testing for toxoplasmosis is that the antibody testing is uncertain. In addition, an ACOG technical bulletin[1] suggests that most women with a first infection acquired during pregnancy, the dangerous one for the fetus, are symptomatic. This observation implies that testing should be limited to patients with symptoms, which flies in the face of most other observations in which only 10% of the pregnant population acquiring toxoplasmosis will have symptoms.[8]

Despite the fact that testing for toxoplasmosis is difficult, it should be offered to the pregnant woman. If a primary infection is suspected, the pregnant woman has options that will decrease her chances of delivering a severely infected newborn. If an acute infection is discovered early in pregnancy, the patient has the choice of pregnancy termination or treatment with the chemotherapeutic agents spiramycin and sulfa to lower the overall incidence of newborn infection and serious CNS pathology.[9] Those patients who are susceptible, i.e., antibody negative, can be given advice that will lower the risk of acquiring the infection,[10] especially to avoid the ingestion of raw meat and unwashed vegetables and intimate contact with hunter cats. Since asymptomatic latent toxoplasmosis can become a threat to anyone subsequently acquiring HIV, such counseling is a worthwhile public-health endeavor for all women who are antibody negative, whether they are pregnant or not. The pitfalls of diagnosing acute toxoplasmosis infection during pregnancy should be recognized by every American obstetrician. The IgM test for toxoplasmosis, done in most commercial laboratories, can detect antibody persisting for long periods, more than 1 year in some instances.[11] Positive-antibody testing for toxoplasmosis prior to pregnancy is a distinct help to the obstetrician. It eliminates the risk to the fetus during pregnancy and avoids the interpretation of IgM test results. If pre-pregnancy testing has not been done for a pregnant patient who is IgM positive for toxoplasmosis, an amniocentesis can be performed with either culture or polymerase chain reaction (PCR) testing for *Toxoplasma gondii*.[12] This approach is more sensitive than umbilical blood sampling and subsequent testing for IgM antibody in fetal blood, which are positive in only 21% of the infected fetuses.[9]

### OTHER

### Measles

Testing for the presence of measles antibody should be done because it can trigger preventive medical intervention. Measles (variola) is not a known teratogen, but primary infections in pregnant adults can induce a higher-than-expected rate of premature labor and delivery.[13] My concern about measles was accentuated by the drop in measles immunizations in the United States during the 1980s and the resulting high number of pregnant women in the 1990s who are measles susceptible. When identified by serologic testing, these patients can be immunized in the postpartum period, for this vaccine is a live virus.

### Varicella

Testing for varicella should be part of prenatal care. For the susceptible adult, an acute infection can cause serious pulmonary infections and even death.[14] In addition, a fetus occasionally gets infected in utero with CNS and limb defects. The susceptible pregnant woman exposed to varicella should receive intravenous (IV) acyclovir if there are signs or symptoms of pneumonia.[15] Frequent sonographic studies of the newborn should be done to detect any fetal abnormalities. In the future, postpartum susceptible women can be given the just-released varicella vaccine which is well tolerated by adults.

### Group B Streptococcus (GBS)

GBS can cause early onset sepsis in the newborn, with death or CNS damage in survivors of post-delivery antibiotic treatment. This problem is seen more frequently in women who deliver premature babies,[16] and newborn outcome is improved by the intrapartum administration of antibiotics compared with postpartum administration.[17] To address the issue of antepartum prophylaxis, the American College of Pediatricians has recommended that vaginal and rectal cultures for GBS be done at 26–28 weeks gestation, with high-risk patients who are culture positive at the time of screening being given antepartum antibiotic prophylaxis.[18] These high-risk patients include women with preterm labor, premature rupture of the membranes, and prolonged rupture of the membranes in term births and women with multiple gestations. I believe this strategy is hopelessly flawed because the GBS colonization in pregnant women is a skip phenomenon, not a constant. Indeed, one longitudinal study of pregnant women found that only 51% of those who were colonized at term had been culture positive when screened in the first trimester.[19] This means that some women treated on the basis of the suggested midtrimester culture will be culture negative and some women who are culture negative at 26–28 weeks but culture positive at the time of delivery will not be treated. At present, the most logical approach is the one suggested by Minkoff and Mead:[20] High-risk patients are cultured at the time of admission. If they go into labor before cultures are available or if they are culture positive before labor ensues, they should be treated with antibiotics prior to delivery. There should be one caveat for whatever strategy is chosen by obstetricians. Based upon our current state of knowledge, we cannot prevent all acute onset GBS infections. Even treating every patient admitted in labor with antibiotics may not prevent newborn infection and death.[21] The ultimate answer to this serious newborn problem will be the development of a protective vaccine or more rapid, accurate microbiologic screens for the presence of this organism.

### Neisseria gonorrhoeae

*N. gonorrhoeae* is a pathogen that, in the pregnant woman, has been associated with premature labor and delivery.[22] Screening for this pathogen should be done at the initial obstetrical-care contact. If the screening test is positive, both the patient and her male sexual partner should be treated.

### Chlamydia trachomatis

The significance of *C. trachomatis* infection for the pregnant woman has been questioned. Although some studies have shown a relationship between positive cultures and pregnancy complications,[23] other studies have not shown this relationship.[24] Recent studies by Witkin et al.[25] have demonstrated a broad impact of infection with *C. trachomatis* on reproductive processes. It seems clear from these sophisticated studies that tubal damage due to infection with this organism is an autoimmune phenomenon. The human host's allergic response to the heat-shock protein of *C. trachomatis* triggers a response with subsequent infection with chlamydia or other genital-tract organisms, in which the body cannot distinguish between the heat-shock protein of chlamydia and normal body protein. The resulting tubal damage causes infertility[26] or an increased incidence of tubal pregnancy.[27] Some recent studies of infertile couples who either conceived naturally[28] or through the in vitro process[29] have shown an increased rate of abortion. Therefore, as a minimum, first-trimester screening for *C. trachomatis* should be done with the most sensitive screening technique, the PCR,[30] and those patients who are positive plus their sexual partners should be treated with either erythromycin or amoxicillin.[31] There is an additional problem near delivery. Those women who are carriers of *C. trachomatis* may infect their newborns with a resultant conjunctivitis not cured by local antibiotic preparations. I believe that all pregnant women should be screened for *C. trachomatis* in the third trimester and those women who are positive treated with either systemic erythromycin or amoxicillin.[31]

### Tuberculosis

There has been a resurgence of tuberculosis in the United States in the past decade. The increase in the number of people living in poverty, the emergence of a large number of homeless people, and the entry to the United States of large numbers of immigrants from the Caribbean and Southeast Asia with a high proportion of tuberculosis have increased the number of pregnant women with active tuberculosis. Among urban poor pregnant patients, a minimum standard should be universal skin testing, with chest roentgen examination for all those who are skin-test positive. If the chest X-ray is normal, these women with no history of treatment or vaccination (BCG, Quad Pharmaceuticals, Inc., Indianapolis, IN) should be treated for a minimum of 6 months with isoniazid (INH, CIBA Pharmaceutical Company, Summit, NJ).[32] Women with active pulmonary lesions should be treated with a combination of systemic agents based upon antibacterial susceptibility studies. To prevent fetal eighth-nerve toxicity, aminoglycosides should be avoided.[33] There is an additional problem for physicians planning tuberculosis screening in pregnancy. A pregnant woman with acquired immunodeficiency syndrome (AIDS) who has an active tuberculosis infection can be skin-test negative.[34] A chest X-ray is the minimum requirement for screening in a patient who is HIV positive. If the HIV-positivity rate approaches 1% in a pregnant population, I believe that routine chest X-rays with abdominal shielding should be done for all prenatal patients.

### RUBELLA

Rubella is the grandfather of the STORCH[5] pathogens because of the relationship between maternal infection and newborn abnormalities noted by Gregg[35] in the 1940s. There were 2 major breakthroughs in the 1960s that markedly lowered the incidence of newborn infection due to this organism. The virus was isolated and a vaccine was developed. Preschool immunization of children in the United States has resulted in enough herd immunity that no epidemics of rubella have been seen since the clinical introduction of the vaccine. Despite this immunization program, not every pregnant woman is immune. Ideally, a woman should be screened before her first pregnancy. If she is immune, this fact should be noted for any subsequent pregnancy. Susceptible women can be immunized. For reasons that I do not understand, adult women have a much higher incidence of arthritic symptoms following rubella immunization than adult men.[36] All female patients should be warned of this possibility before receiving the vaccine. In most cases, this complication is a transient problem that is easily treated with antiprostaglandins, although the symptoms may persist for months. An informed consent with this information should be obtained to assure the patient's knowledge of this possibility.

### CYTOMEGALOVIRUS

Cytomegalovirus screening is the source of my second-biggest bone of contention with the ACOG technical bulletin,[1] ranking just below toxoplasmosis. I agree with their assessment of the problem. Cytomegalovirus is the most common cause of newborn infection, infecting nearly 1% of all newborns.[37] From here on, we diverge, for the ACOG technical bulletin states that, since nothing can be done, no testing should be done. This flies in the face of reality. It seems clear from prior studies in Alabama that a pre-pregnancy infection with cytomegalovirus confers some protection to the fetus; both the number and severity of infections are reduced.[38] This information is valuable for the mother and the obstetrician, and a recent vs. old infection can be ascertained by serial determinations of IgG and IgM. The cytomegalovirus-susceptible patient should be counseled by her obstetrician. Hospital nurses in high-risk areas for the acquisition of cytomegalovirus can markedly reduce their rate of cytomegalovirus infection by washing their hands thoroughly after each patient encounter.[39] For the susceptible pregnant woman who is not a nurse, the biggest source of infection is young children in day-care centers[40] or in school. These infected children shed virus in their oral secretions and urine. These susceptible pregnant mothers-to-be should be advised to avoid kissing these children and to wash their hands thoroughly after they change a diaper. Pregnant women can acquire cytomegalovirus through blood transfusions, so blood banks should use cytomegalovirus-seronegative blood for women who are susceptible.[41] Various treatments including vaccines[42] and acyclovir[43] have been tested in immunosuppressed nonpregnant patients. Perhaps such interventions will be tested in pregnant women in the future.

### HERPES

### Herpes (H[1])

My thinking about herpes infections in the newborn has undergone major revisions in the past decade because of an explosion of new information. In the past, I focused upon the patient with a history of genital herpes and used weekly vaginal cultures for herpes from 36 weeks gestation until term in this population to determine whether vaginal delivery or cesarean section is indicated.[44] A number of observations have made me abandon this strategy. Studies have determined that a primary infection during pregnancy is much more dangerous to the fetus than a recurrent infection;[45] only 10% of women with HSV-2 infection as determined by positive-antibody status will ever have any signs or symptoms of a genital herpes infection;[46] 70% of the newborn infections caused by herpes occur in women who either have no history of genital herpes infection or, if they have a history, are asymptomatic at the time of delivery;[47] and the weekly genital culture for herpes is not predictive when the patient returns in labor.[48] Because of these findings, I no longer do weekly cultures for herpes near term. I pay close attention to any patient symptomatology suggesting that a genital herpes outbreak is about to occur and examine the patient for genital lesions when she is admitted in labor. If either symptomatology or lesions are present, I do an immediate cesarean section. Finally, in a patient with a history of genital herpes, I do not use scalp electrodes, thus avoiding an invasive procedure in a woman who could be shedding herpes virus asymptomatically.[49] There are case reports of scalp infections due to the use of invasive technology[50] and this risk is totally avoidable. Not every investigator agrees with this stand, but a group of discriminating Canadian physicians supports this position.[51]

### Hepatitis B ($H^2$)

The incidence of hepatitis B infection in the United States has increased markedly in the past decade. There are a number of obvious contributors to this alarming trend: an increase in poverty; continued dissolution of the family unit among the urban poor, particularly black Americans; an influx of immigrants from the Caribbean and the Far East where the incidence of infection with this virus is high; increased sexual contacts without the use of barrier contraceptives, for it is a sexually transmitted disease; and the sharing of needles among IV drug users. This increase in the incidence of hepatitis B infections in the United States has been accompanied by an increase in hepatitis B infection in the newborns of women who are antigen positive for hepatitis B. An early strategy is to restrict screening to high-risk mothers in an effort to lower health-care costs. Articles still appear in our literature evaluating the cost effectiveness of universal screening for the hepatitis B antigen. The fact of the matter is that restricting screening to high-risk patients means that a good number of hepatitis B antigen-positive mothers will be missed. Since antepartum detection and postpartum treatment of the newborn with hepatitis B immune globulin and hepatitis B vaccine largely eliminate newborn infection[52] and avoid subsequent cirrhosis and liver cancer, the choice to me is clear. There should be universal antepartum screening for the hepatitis B antigen. Susceptible patients can be immunized postpartum.

### HIV ($H^3$)

Screening for the presence of HIV antibody has been politicized in this country. As a result, it is hard to determine appropriate medical policy because the incidence of infection is unknown and patients can refuse to have testing. For the obstetrician practicing in Massachusetts, important anonymous data are available. All cord bloods are tested anonymously for HIV antibodies, which gives the obstetrician important baseline data. This information makes it possible to know the incidence of maternal HIV antibody-positive women in the obstetrical population for a given time frame. There are major differences in frequency, with a higher incidence seen in inner-city hospitals than in rural areas.[53] In addition, I have been impressed that, on our own obstetrical service at the New York Hospital, only half of the HIV-positive women discovered by anonymous cord-blood sampling have been identified by obstetricians in assessing the high-risk activity of patients. For this reason, I would stress widespread testing in any obstetrical population in which the positive HIV-antibody incidence nears or exceeds 1%. How do you convince women to be tested? Although newborn infection with HIV ranges from 20 to 60% in some studies,[54] the knowledge of a positive HIV-antibody test infrequently results in a decision to terminate the pregnancy in these urban poor women. Instead, prevention measures can be stressed. These patients may be candidates for zidovudine (AZT) prophylaxis during pregnancy and should be screened for *Pneumocystis carinii* pneumonia, tuberculosis, and syphilis. A recent study suggests that AZT prophylaxis in pregnancy lowers the risk of HIV acquisition by the newborn.[55] In addition, appropriately directed antibiotic therapy should increase their number of symptom-free days. The knowledge that a pregnant woman is HIV-antibody positive should influence intrapartum management. Since some of these women will shed HIV in their vaginal secretions, a fetal scalp electrode and fetal scalp sampling should not be employed because either procedure breaks the skin barrier and exposes the newborn to infection.

### HPV ($H^4$)

Obstetricians are at an early phase on the learning curve of an appropriate response to HPV infections. Some important bits of information are available. One study of sexually active female college undergraduates, using a PCR technique, found that 46% had been exposed and infected with this family of viruses.[56] In addition, some strains of this virus have been associated with an increased incidence of cervical carcinoma.[57] The virus has not been grown in clinical-laboratory tissue cultures, and identification to date has been dependent upon pathologic tissue changes and detection of DNA for certain strains of HPV. The pathologic interpretation varies among pathologists, and the DNA probe is limited to a small number of HPV strains. A confirmation of the presence of the virus in cervical tissue usually leads to some type of ablative procedure postpartum. Papillomatosis of the vocal cords of newborns has been reported. Fortunately, this complication has been a rare event in newborns and no studies have indicated that cesarean sections should be performed in such women.

### Human Parvovirus ($H^5$)

An acute infection with this virus can cause intravascular hemolysis. In the fetus, the subsequent anemia can cause nonimmune hydrops which, if untreated, can result in fetal death.[58] Fortunately, maternal infections with human parvovirus are rare and few susceptible newborns develop intrauterine anemia.[59] For the few affected fetuses, a diagnosis of intrauterine anemia can be made by intrauterine fetal blood sampling, and these anemic infants can be effectively treated with intrauterine blood transfusions.

### CONCLUSIONS

This overview of scientific preventive care for the pregnant woman is free of any political considerations. As advocates for the best care for women, we should endorse all of these prenatal preventive measures and put them into practice.

### REFERENCES

1. American College of Obstetricians and Gynecologists: Perinatal viral and parasitic infection. Tech Bull 177: 1–7, 1993.
2. Centers for Disease Control: Congenital syphilis—United States, 1985–85. JAMA 256:3206–3208, 1986.
3. Centers for Disease Control: Relationship of syphilis to drug use and prostitution—Connecticut and Philadelphia, Pennsylvania. MMWR 37:755–764, 1988.
4. Berkowitz KM, Stampf K, Baxi L, et al.: False negative screening tests for syphilis in pregnant women. N Engl J Med 322:270–271, 1990.
5. Centers for Disease Control: Guidelines for the prevention and control of congenital syphilis. MMWR 37:S1–S13, 1988.
6. Musher DM: Syphilis, neurosyphilis, penicillin, and AIDS. J Infect Dis 163:1201–1206, 1991.
7. Kelin VR, Cox SM, Mitchell MD, et al.: The Jarisch-Herxheimer reaction complicating syphilotherapy in pregnancy. Obstet Gynecol 75:375–380, 1990.
8. Wilson CB, Remington JS: What can be done to prevent congenital toxoplasmosis? Am J Obstet Gynecol 138:357–363, 1980.
9. Daffos F, Foreister F, Gapella-Paulovsky M, et al.: Prenatal management of 746 pregnancies at risk for congenital toxoplasmosis. N Engl J Med 318:271–275, 1988.
10. Foulon W, Naessens A, Lauwers S, et al.: Impact of primary prevention on the incidence of toxoplasmosis during pregnancy. Obstet Gynecol 72:363–366, 1988.
11. Brooks RG, McCabe RE, Remington JS: Role of serology in the diagnosis of toxoplasmosis lymphadenopathy. Rev Infect Dis 9:755–782, 1987.
12. Groover CM, Thulliez P, Remington JS, et al.: Rapid prenatal diagnosis of congenital toxoplasma infection by using polymerase chain reaction and amniotic fluid. J Clin Microbiol 28:2297–2301, 1990.
13. Siegel M, Fuerst HT: Low birthweight and maternal viral diseases. A progressive study of rubella, measles, mumps, chickenpox, and hepatitis. JAMA 197:680–684, 1966.
14. Balducci J, Rodis JF, Rosengren S, et al.: Pregnancy outcomes following first-trimester varicella infection. Obstet Gynecol 79:5–6, 1992.
15. Smego RA Jr, Asperilla MO: Use of acyclovir for varicella pneumonia during pregnancy. Obstet Gynecol 78: 1112–1116, 1991.
16. Baker CJ, Barrett FF: Transmission of group B streptococci among parturient women and their neonates. J Pediatr 83:919–923, 1973.
17. Pyati SP, Pildes RS, Jacobs NM, et al.: Penicillin in infants weighing two kilograms or less with early onset group B streptococcal disease. N Engl J Med 308:1383–1389, 1983.
18. Committee on Infectious Diseases in Fetus and Newborn: Guidelines for prevention of group B streptococcal (CBS) infection by chemoprophylaxis. Pediatrics 90:775–778, 1992.
19. Lewin EB, Amstey MS: Natural history of group B streptococcus colonization and its therapy during pregnancy. Am J Obstet Gynecol 139:512–515, 1981.
20. Minkoff H, Mead P: An obstetric approach to the prevention of early onset group B β-hemolytic streptococcal sepsis. Am J Obstet Gynecol 154:973–977, 1986.
21. Hamoudi AC, Hamoudi AB: Fetal group B streptococcal pneumonia in neonates. Effects of antibiotics. Am J Clin Pathol 76:823–826, 1981.
22. Handsfield HH, Hudson WA, Holmes KK: Neonatal gonococcal infection. JAMA 225:697–701, 1973.
23. Martin DH, Koutsky L, Eschenbach DA, et al.: Prematurity and perinatal mortality in pregnancies complicated by maternal *Chlamydia trachomatis* infections. JAMA 247: 1585–1588, 1982.
24. Harrison HR, Alexander ER, Weinstein L, et al.: Cervical *Chlamydia trachomatis* and mycoplasmal infections in pregnancy. JAMA 250:1721–1727, 1983.
25. Witkin SS, Jeremias J, Toth M, et al.: Cell mediated immune response to the recombinant 57-kDa heat shock protein of *Chlamydia trachomatis* in women with salpingitis. J Infect Dis 167:1379–1383, 1993.

26. Brunham RC, McLean IW, Binns B, et al.: *Chlamydia trachomatis:* Its role in tubal infertility. J Infect Dis 152: 1275–1282, 1985.
27. Brunham RC, Binns B, McDowell J, et al.: *Chlamydia trachomatis* infection in women with ectopic pregnancy. Obstet Gynecol 67:722–726, 1986.
28. Witkin SS, Ledger WJ: Antibodies to *Chlamydia trachomatis* in sera of women with recurrent spontaneous abortion. Am J Obstet Gynecol 167:135–139, 1992.
29. Licciardi F, Grifo JA, Rosenwaks Z, et al.: Relation between antibodies to *Chlamydia trachomatis* and spontaneous abortion following in vitro fertilization. J Assist Reprod 9:207–210, 1992.
30. Witkin SS, Jeremias J, Toth M, et al.: Detection of *Chlamydia trachomatis* by the polymerase chain reaction in the cervices of women with acute salpingitis. Am J Obstet Gynecol 168:1438–1442, 1993.
31. Cromblehome WR, Schacter J, Grossman M, et al.: Amoxicillin therapy for *Chlamydia trachomatis* in pregnancy. Obstet Gynecol 75:752–756, 1990.
32. Snider DE: Pregnancy and tuberculosis. Chest 86:10S–13S, 1984.
33. Glassroth J, Rubins AG, Snider DE Jr: Tuberculosis in the 1980s. N Engl J Med 302:1441–1450, 1980.
34. Pitchenik AE, Fertel D, Blocker AB: Mycobacterial disease: Epidemiology, diagnosis, treatment, and prevention. Clin Chest Med 9:425–441, 1988.
35. Gregg NM: Congenital cataract following German measles in the mother. Trans Aust Coll Ophthalmol 3:35–39, 1941.
36. Howson CP, Katz M, Johnston RB Jr, et al.: Chronic arthritis after rubella vaccination. Clin Infect Dis 15: 307–312, 1992.
37. Yow MD, Demmler GJ: Congenital cytomegalovirus disease—20 years is long enough. N Engl J Med 326:702–703, 1992.
38. Fowler KB, Stagno S, Pass RF, et al.: The outcome of congenital cytomegalovirus infection in relation to maternal antibody status. N Engl J Med 326:663–667, 1992.
39. Balfour CL, Balfour HH Jr: Cytomegalovirus is not an occupational risk for nurses in renal transport and neonatal units: Results of a prospective surveillance study. JAMA 256:1909–1914, 1986.
40. Murph JR, Baron JC, Brown CK, et al.: The occupational risk of cytomegalovirus infection among day-care providers. JAMA 265:603–608, 1991.
41. Bowden RA, Sayers M, Flournoy N, et al.: Cytomegalovirus immunoglobulin and seronegative blood products to prevent primary cytomegalovirus infection after marrow transplant. N Engl J Med 314:1006–1010, 1986.
42. Plotkin SA, Starr SE, Friedman HM, et al.: Effect of Towne live vaccine on cytomegalovirus disease in patients receiving renal transplants. Ann Intern Med 114:525–531, 1991.
43. Balfour HH, Chace BA, Stapleton JJ, et al.: A randomized placeob-controlled trial of oral acyclovir in recipients of renal allografts. N Engl J Med 320;1381–1387, 1990.
44. Grossman HJ III, Wallen WC, Sever JL: Management of the herpes simplex virus during pregnancy. Obstet Gynecol 58:1–4, 1981.
45. Brown ZA, Vontuer LA, Benedetti J, et al.: Effects on infants of a first episode of genital herpes during pregnancy. N Engl J Med 317:1246–1251, 1987.
46. Breinig MK, Kingsley LA, Armstrong JA, et al.: Epidemiology of genital herpes in Pittsburgh: Serologic, sexual, and racial correlates of apparent and inapparent herpes simplex infections. J Infect Dis 162:299–305, 1990.
47. Whitley RJ, Corey L, Arvin A, et al.: Changing presentation of herpes simplex virus infection in neonates. J Infect Dis 158:109–116, 1988.
48. Prober CG, Hensleigh PA, Boucher FD, et al.: Use of routine viral cultures at delivery to identify neonates exposed to herpes simplex virus. N Engl J Med 318:887–891, 1988.
49. Ledger WJ: Neonatal herpes simplex infection. N Engl J Med 319:872, 1988.
50. Whitley RJ, Nahmias AJ, Visuntine AM, et al.: The natural history of herpes simplex virus infection of mother and newborn. Pediatrics 66:489–494, 1980.
51. Eason E, Banjamin A: Preventing neonatal herpes. N Engl J Med 327:647–648, 1992.
52. Immunization Practices Advisory Committee: Recommendations for protection against viral hepatitis. MMWR 34:313–335, 1985.
53. Hoff R, Berandi VP, Weiblen BJ, et al.: Seroprevalence of human immunodeficiency virus among childbearing women. N Engl J Med 318:525–530, 1988.
54. Lindsay MK, Peterson HB, Feng TI, et al.: Routine antepartum human immunodeficiency virus infection screening in an inner city population. Obstet Gynecol 74:289–294, 1989.
55. National Institutes of Health: NIH Clinical Alert, 1994.
56. Bauer HM, Ting Y, Greer CE, et al.: Genital human papillomavirus infection in female university students as determined by a PCR-based method. JAMA 265:472–477, 1991.
57. Reeves WC, Brinton LA, Garcia M, et al.: Human papillomavirus infection and cervical cancer in Latin America. N Engl J Med 320:1437–1441, 1989.
58. Peters MT, Nicolaides HK: Cordocentesis for the diagnosis and treatment of human fetal parvovirus infection. Obstet Gynecol 75:501–504, 1990.
59. Boden E, Eriksson A, Rylander E, et al.: Clinical characteristics of papillomavirus vulvovaginitis. Acta Obstet Gynecol Scand 67:147–151, 1988.
